# Antiradical Potential of Food Products as a Comprehensive Measure of Their Quality

**DOI:** 10.3390/foods11070927

**Published:** 2022-03-23

**Authors:** Victor Gorbachev, Maria Klokonos, Sherzodkhon Mutallibzoda, Svetlana Tefikova, Olga Orlovtseva, Natalia Ivanova, Galina Posnova, Daria Velina, Igor Zavalishin, Mars Khayrullin, Elena Bobkova, Elena Kuznetsova, Alla Vorobeva, Dmitry Vorobyev, Igor Nikitin

**Affiliations:** 1 Department of Biotechnology of Food Products from Plant and Animal Raw Materials, K.G. Razumovsky Moscow State University of Technologies and Management (The First Cossack University), 73 Zemlyanoy Val, 109004 Moscow, Russia; genetic2@yandex.ru (V.G.); m.klokonos@mgutm.ru (M.K.); mutallibzoda@bk.ru (S.M.); s.tefikova@mgutm.ru (S.T.); o.orlovtseva@mgutm.ru (O.O.); n.ivanova@mgutm.ru (N.I.); g.posnova@mgutm.ru (G.P.); kattim67@gmail.com (D.V.); 2 Department of Automated Control Systems, K.G. Razumovsky Moscow State University of Technologies and Management (The First Cossack University), 73 Zemlyanoy Val, 109004 Moscow, Russia; 3 Research Department, K.G. Razumovsky Moscow State University of Technologies and Management (The First Cossack University), 73 Zemlyanoy Val, 109004 Moscow, Russia; m.hairullin@mgutm.ru (M.K.); vica3@yandex.ru (E.B.); e.kuznecova@mgutm.ru (E.K.); vorobevaav@mgutm.ru (A.V.); d.vorobyev@mgutm.ru (D.V.)

**Keywords:** antiradical activity, DPPH, bread, confectionery, milk, eggs, meat, fish, cereals, drinks, wild herbs, shelf life, biologically active substances

## Abstract

Antiradical potential (ARP) is an important measure of food safety. In addition, it directly or indirectly affects the rate of occurrence of a number of human pathologies. Using a photocolorimetric analysis of DPPH (2,2-diphenyl-1-picrylhydrazyl) solutions, we estimated the antiradical potential of food raw materials, food concentrates, biologically active substances, and wild plants. We conducted approximately 1500 analyses of almost 100 food products selected from 11 food groups: vegetables, milk, meat, fish, cereals and bread, drinks (including tea and coffee), etc. With a confidence interval (CI) of 95%, the average values for animal products range from 15.87 to 18.70 ascorbic acid equivalents per gram of dry matter. For plant materials, the range is 474.54–501.50 equivalents when wild herbs are included and 385.02–408.83 equivalents without taking herbs into account. The antiradical potential of the biologically active substances we studied ranged from 706.84 to 847.77 equivalents per gram of dry matter, which makes it possible to use some of the components to repair products with low ARP values, for example, bread and baked goods, confectionery, milk and dairy products, carbonated drinks, and juice. In this study, a low ARP value is associated with a reduction in the shelf life of products and a deterioration in their organoleptic properties; therefore, we propose using ARP as an important reference for describing the quality of food products and raw food materials.

## 1. Introduction

The annual expansion of the production of highly refined and processed food products is noted by a number of authors as one of the most urgent problems facing the global food industry [1,2,3,4,5,6,7].

Most scholarship devoted to the processing of food products can be divided into three groups: research assessing the anthropogenic impact of production on the biosphere [8,9]; studies focused on the medical, legal, and economic problems caused by the consumption of refined foods [1,4,5,7]; and work that explores the problems of individual consumers in assessing the quality of food products [1].

When analyzing food products, the impact on human health is most often the focus of assessment due to the potentially propathological effect of refined food products [3,10]. The need for affordable and convenient foods in fast-growing urban agglomerations is pushing manufacturers to use “simple and cheap” sources of raw materials such as sugars and fats. Although this approach makes food more energy-intensive, it also contributes to nutritional imbalance and the loss of a number of nutrients, provoking a number of pathological processes in the human body, such as lipid peroxidation, lipidemia, free radical pathologies, obesity, diabetes, cardiovascular disease, and other problems. [4,7,8,10,11,12,13].

The problem of expanding the market for refined products takes the form of a choice between “healthy” and “harmful” foods. So, for example, researchers studying the market in India, Europe, Latin America, and Russia demonstrated a high level of consumption of processed food products (at least 50% of consumers) [14,15,16,17].

In addition to the loss of some nutrients and the decline in nutritional properties relevant to consumers, another non-obvious consequence is manifested during refining: a decrease in the shelf life of the product. This leads to the need for additional spending on measures to protect the food system from risks, including autoxidation [2,18].

Although the topics listed above are interconnected, the shelf life, the chemical composition, and the nutritional integrity of food are of particular importance for the average consumer as indicators of its quality [18]. However, to date, no unambiguous characteristic (or system of characteristics) has been developed that could be used for a comprehensive description of all the above-listed properties. Potentially, this role can be played by the antiradical potential (hereinafter ARP) of food, which indirectly characterizes its chemical composition and can also be used to assess the duration and stability of food storage [2,4,5,18].

Several methods are used to assess the ARP; however, the method of measuring the optical density of solutions of the stable radical DPPH has become most widely used [19]. No significant technical shortcomings for this method have been described in scientific publications. However, there is a methodological disadvantage associated with the fact that the data presented in various studies are almost impossible to compare with each other. Comparisons can only be made within each specific study. A partial solution would be to carry out large-scale analyses of foodstuffs from various food commodity groups, primarily to obtain activity results on a single “scale” of measurement.

Thus, the purpose of our study is to analyze the ARP data of food systems from various food product commodity groups and evaluate the possibility of using this data to describe the quality of food products and the relationship of this parameter to their shelf life.

## 2. Materials and Methods

### 2.1. Food Materials

Randomly selected food products served as the material for this study. For the analysis, we chose types of food products that are frequently used in the food industry. 

For the analysis, we evaluated food products, biologically active substances (hereinafter dietary supplements), and wild plants from the following categories: bread, bakery and confectionery products, seaweed and wild plants of the northeast of Russia, vegetable raw materials (including fresh-frozen) of industrial production, mushrooms, meat and meat products, fish and canned fish, cereals, milk and its products, amino acids, protein hydrolysates, fatty acids and vitamins, as well as drinks. In total, 11 summary tables were compiled that characterize the ARP of each of the listed product groups. If a table does not indicate the place or country of production, then by default, it is Russia, with the exception of certain rare cases where it was not possible to obtain such information. Weighed samples that were subjected to heat treatment were irradiated with high-frequency radiation (microwave radiation) to a state of visual readiness (for products of animal origin) or softness (for products of plant origin), and moisture content was also determined for each sample. All wild plants and algae were collected, identified, and processed by the authors themselves.

### 2.2. Extract Preparations

Samples of products were weighed for extraction and also for determination of dry weight after drying them at 110–115 °C for 24 h. Samples were roughly crushed with a scalpel (with the exception of already crushed products) and poured into pharmaceutical bottles with a dense, ground-in soft rubber stopper. The solvent used was 96% ethyl alcohol at a dry matter to solvent ratio of approximately 1:10 (with the exception of the algae, which were analyzed earlier and added to the study for comparison). The samples were infused at −20 °C for a week with occasional stirring. To eliminate the autoxidation of antiradical fractions, the unfilled volume in the bottles was purged with a fivefold volume of carbon dioxide, which was fed into the bottle through a long medical probe, and excess air was removed from the bottle through a second probe of shorter length. 

### 2.3. ARP Measurement

The measurements were carried out on an optical photocolorimeter, taking into account the wavelength transmission width from 515 to 565 nm, with a DPPH absorption maximum of 517 nm. To measure the optical density of the products, a solution of DPPH in 96% ethyl alcohol was used, with a concentration of 1.27 × 10^−4^ mol/liter, which was stored in a dark place at −20 °C.

The DPPH solution was previously calibrated using a series of dilutions of ascorbic acid from 1 × 10^−^^6^ to 1 × 10^−^^8^ mol/liter. Taking into account the data obtained, a graph of 50% discoloration of the solution was created, which amounted to 4.24 × 10^−^^7^ mol per reaction. On the basis of these calibrations, the relationship between the extinction drop and the quantity of ARP in a substance was determined, which was ≈0.0062 per cm/eq × 10^−^^2^ (per centi–equivalent, further ceq or eq × 10^−^^2^). Based on the above data and the differences in the optical density of the analyzed samples, the ARP values were calculated, factoring in the weight of the dry matter taken for analysis as well as the degree of dilution of the extract. A CI of 95% was calculated for the obtained values. 

The analysis was carried out using a negative control (without discoloration and with the addition of 0.5 mL of pure alcohol) and a positive control (with the addition of 0.5 mL of a 0.01 molar alcohol solution of ascorbic acid). The volume of the reaction mixture was 2.5 mL, of which 2 mL was for the calibrated DPPH solution and 0.5 mL for the analyzed extract, as previously described by us [20]. If the bleaching occurred completely and the extract showed high antiradical activity, then the sample was diluted 1, 5, 10, and 50 times. The exposure time of the solution was 30 min in a dark place. All samples were analyzed 5 times, while the values were rechecked 3 times, taking into account the instrument error rate of 0.3%. In accordance with the recommendations given earlier in the literature [19], μmol of ascorbic acid was taken as 1 equivalent. All ARP values are given on dry weight. Statistical calculations (using mean values with a 95% CI) were carried out in MS Excel 2010.

## 3. Results and Discussion

We begin by discussing the data for each food commodity group separately. In view of the importance of bread and bakery products in the human diet, we begin the discussion with them. The results are presented in Table 1. We note that bread and bakery products demonstrate the lowest ARP values of all products studied.

A comparison within Table 1, however, shows a clear upward trend in the average ARP values in the following row: products made from pure wheat flour < from mixed rye–wheat flour without additives < from mixed rye–wheat flour with vegetable additives < from rye flour. At the same time, the first two categories do not statistically differ from each other and overlap with 95% confidence intervals. At first examination, the question arises as to why cannabis-infused biscuit extracts show low ARP values. We believe that the cause is complex and will be discussed below. In the meantime, we note three points necessary for understanding the obtained ARP values: the chemical stability of food system components, the refining of the composition of the product during the technological processing of food raw materials, and the speed and mechanism of the interaction of food components with already existing environmental oxidants and free radicals.

Leaving the table on bakery products, we progress to a more detailed discussion involving Table 2, which contains data on the ARP of some commonly used components in the food industry, including vitamins and hydrolysates as well as other biologically active substances and additives (hereinafter BAS/BAA).

In view of the large amount of data contained in this table, we offer the following analysis. All substances can be grouped into three categories: proteins, their hydrolysates, pure amino acids, and their derivatives (the top portion of the table); lipophilic substances such as lecithins, tocopherols, and thioctic and fatty acids (the middle portion of the table); and hydrophilic substances such as B vitamins, bioflavonoids, and mineral components (the lower portion of the table).

This table includes the substances with the highest obtained values of ARP in our study, the first of which is acetylcysteine (NAC), a product used both in the food and pharmaceutical industries. This substance belongs to the group of cysteine derivatives. This group also includes the peptide glutathione, known primarily for its role in biochemical recovery processes that greatly deplete its pool in cases of toxic poisoning [21]. Despite the fact that glutathione can be synthesized in humans and animals, it is not stable in the gastrointestinal tract; it is destroyed, which makes it necessary to replenish its pool through the use of cysteine itself, its derivatives, or food enriched with it [3,12,21].

It is known that all proteinogenic amino acids have a different molecular structure, which affects their solubility and the rate of interaction during redox reactions. According to the features of their side fragments, amino acids are classified either as ionogenic (seven in total) or non-ionic, which are in turn divided into two groups: those with polar side chains (four structures) and those with non-polar side chains (nine structures) [12]. 

Previous attempts were made to establish the ARP of amino acids, but methodically these efforts were limited by a number of difficulties. Some authors proposed that the activity of amino acids is associated only with the presence or absence of one or another functional group in the molecule [22,23,24,25]. For this reason, we analyzed representatives of all three groups; our results did not confirm the previously suggested theory. For example, cysteine derivatives showed the highest ARP compared to all other groups of amino acids, despite the fact that the structure of acetylcysteine contains sulfur along with methionine [24,25]; its ARP values were very low, at the level of statistical error, and are not shown. On the other hand, monosodium glutamate showed one of the lowest values among amino acids, demonstrating the heterogeneity of ARP values in this group of substances.

We, along with a number of other authors, did not expect such a large diversity in the obtained values; the existence of different ARP mechanisms in different groups of amino acids has been suggested to explain this [26]. Mathematical modeling previously published showed that the ARP of amino acids is affected by three factors: the molecular weight, the ionogenicity, and the hydrophobicity or hydrophilicity of the molecule as a consequence [26]. At the same time, we found that different groups of amino acids differ in their oxidative process mechanism. If there are radicals or non-polar or pro-oxidative particles (for example, Fe ^+2/+3^ ions) in mixtures, they react faster with non-polar amino acids. However, this feature affects the kinetics of the chemical reaction, and structures with low levels of activity do not acquire additional ARP. In addition, there is chelation of ions with variable valence by low molecular weight peptides and some amino acids (methionine, tryptophan, etc.). This chelation affects the decrease in the prooxidative action of these ions over a long time interval and helps to preserve the quality of food products by a different mechanism, even at a low level of activity (e.g., for methionine) with regard to the capture of free radicals [27].

According to the literature and our own data, protein hydrolysates have a higher ARP than pure protein preparations. For example, it has been previously shown that the reducing ability of proteins increases with the degradation of the molecular weight of the protein [26,28]. In other words, the ARP of the native protein is less (with a reservation about some enzymes) than that of the hydrolysis products of its molecule. As molecular degradation increases, the ARP values also increase until it reaches the maximum value, characteristic of the mixture of individual amino acid molecules included in it.

For our data, a similar pattern is observed. For example, whey protein hydrolyzate is 9.5–10 times more active than wheat gluten protein from bakery products made from premium flour. We attribute this both to an increase in the spatial availability of the amino acid side chains of polypeptides and to an increase in the concentration of C- and N-terminal fragments of polypeptides per volume of reacting mixtures [12,28]. With some exceptions, all studied samples are distributed in the following order according to the increase in their activity: native proteins < less protein-enriched hydrolysates < more protein-enriched hydrolysates < a mixture of molecular amino acids. On average, the values for molecular amino acids are higher than all of the hydrolysates, despite the low values for some group representatives.

The second group presented in Table 2 is the group of lipophilic substances. Primarily it includes fatty acids, phosphatidylinositols as a fraction of lecithins, and lipoic acid. In previous toxicological studies, it was shown that, for example, molecular oxygen is lipophilic and dissolves in lipids 7–10 times better than in water [21,29]. As a result, lipid fractions are subjected to stronger oxidation. In addition to the direct oxidation of the components of lipid membranes of cells (as well as the fatty phase of food systems), oxygen also induces cascades of peroxidation reactions according to the free radical mechanism [12,21]. This leads to the rapid destruction of the carbon skeleton of lipid molecules, the rancidity of fats, and a decrease in the vitamin activity of lipophilic vitamins. As a result, the quality of food products falls, and their shelf life decreases [2,3,4,5,18].

For this reason, both to improve the organoleptic properties of food products and to establish the temporal stability of food systems, it is necessary to introduce additional portions of antioxidants or to use raw materials with initially high rates thereof [2,3]. However, a simple increase in the concentration of vitamins cannot solve this problem, since it is known that vitamins C, B, and E, when mixed in food systems, reduce each other’s stability [3]. At high concentrations, vitamin E also exhibits prooxidative properties, thus increasing the oxidation of other components [30,31]. Perhaps the solution lies in the replacement of a portion of the tocopherols with tocotrienols, but this possibility requires additional research.

In the studied group of lipophilic components, the activity of both individual vitamins (vitamin E and thioctic acid) and membrane lipids (soy lecithins) was tested. The low values for tocopherol are explained by the slow rate of reaction with the free radical DPPH, as well as its limited solubility in 96% ethanol. The exposure time did not exceed half an hour, and not all the activity of tocopherol had time to manifest itself during this period. When the mixture was left for a long time, the activity increased significantly. The “slowness” of this group of vitamins plays an important role in maintaining product quality during long-term storage [2,3].

Another group of substances capable of interacting with radicals is the group of polyunsaturated fatty acids, including those that are a part of the lecithin phosphotidylinositols structure, which, in our opinion, explains the ARP level of this food supplement. As the number of diene structures in fatty acid molecules (or conjugation) increases, their ARP increases while stability decreases. At the same time, ARP also increases in the following series: CLA conjugated lenolic acid (two unsaturated bonds) < arachidonic acid (four bonds) < EPA + DHA (five and six bonds). Attentive analysis of Table 2 shows that the reported values of the phosphatidylinositols are higher (arachidonic acid), but it is worth clarifying here that, as in the case of tocopherols, EPA and DHA cannot fully react within half an hour, but they have a greater potential during long-term incubation. In other words, the activity of arachidonic acid is higher, but ARP is higher in eicosapentaenoic and docosahexaenoic acids due to their greater number of polyene structures [12]. In our opinion, lipoic acid exhibits higher values due to the presence of sulfur in the structure of the molecule, which brings it closer to the cysteine derivatives. 

Continuing the comparison of the data, we examine the third group we established for analysis: hydrophilic polyphenol compounds (flavonoids, anthocyanins, etc.) and hydrophilic vitamins. This group has long been known for its ability to block free radical reactions; they prevent them from occurring, resulting in the name “free radical traps” [3,12,13]. This group includes more than a hundred substances, but their use in the food industry is very limited. Most often, this is due to the use of raw materials that already contain these groups of substances. This group, as we will show below, is quite stable in terms of temperature and can perhaps be successfully used to repair the lost ARP of already prepared foodstuffs, including in mixtures.

To summarize, we note that we can distinguish three types of antiradical activity in food systems: the first is activity that occurs when interacting in a solution of water or hydrophilic solvents; the second is activity in the lipid fraction; the third is activity aimed at blocking prooxidative particles that enhance the course of redox reactions through chelation or other interactions.

We return now to Table 1. The basis of wheat bread is gluten or wheat proteins [32], refined from both seed coats (containing B vitamins) and germ reserve substances (proteins and oils). For rye bread, wholemeal flours (whole grains) are used; this affects the concentration of B vitamins and lipids, which, as shown in Table 2, exhibit a high level of ARP. As a result, a greater quantity of these substances increases the indicators for rye bread in relation to wheat bread. Bread products made from mixed flour occupy an intermediate position. In addition, we note that any addition of vegetable raw materials, such as cranberries to the “Tayozhny” bread, leads to an increase in the ARP values, not only due to the vitamins the vegetable matter contains but also due to the presence of coloring substances such as polyphenols and carotenoids.

On the other hand, there is the question of why hardtacks with the addition of hemp flour have a low value. First of all, the proportion of hemp flour is small and does not exceed a few percent, and thus it cannot shift the values strongly upwards. Secondly, polyunsaturated fatty acids exhibiting antiradical activity (Table 2), as mentioned above, are rapidly oxidized.

Thus, it is necessary to introduce larger portions of substances with ARP into those hardtacks, to replace the refined flour in the recipe with flour of a lower grade, to reduce the shelf life of this food product, or to use inert gas packaging. All of these actions are justified; rancidity, a common defect for bakery and flour-based confectionery products, can have its manifestation delayed by the correction of ARP levels.

Candy products also show a low level of ARP (Table 3). The mean value is higher than that of bakery products primarily due to the analysis results for molasses, cocoa powder, and dark chocolate.

Returning to Table 1, we note that an additional reason for the increase in ARP in rye bread may be the sugary products added to it, such as molasses. This conclusion is made based on the fact that the activity parameters for cane molasses are higher than all the studied values for bakery products (5.3–5.7 times on average). According to Table 3, waffles made from premium wheat flour also have a low level of activity and overlap with the values obtained, for example, for wheat bread. The level of ARP in products such as marmalade made from agar-agar, “Druzhba” halva made from grated peanut seeds, and pink milk chocolate is almost 2 times higher than in waffles. One reason is that algal polysaccharides (contained in agar-agar, which is used in marmalade preparation) are known to have some residual activity [33,34]. Nut seeds and chocolate contain lipophilic vitamins; chocolate also contains a significant amount of plant polyphenols, which explains its ARP [35].

When comparing different types of chocolate, one can trace the upward trend in ARP for this group of products. We justify this by the increase in the proportion of cocoa powder in the respective products: 34% for the milk chocolate and 75% for the dark chocolate. Cocoa butter was not included in our study, since in the literature its ARP values are noted to be much lower than of chocolate itself [35]. We selected expired cocoa powder (with a production date of December 2011) for analysis, primarily to compare the drop in ARP indicators after such a long shelf life. Since the packaging was not broken, the product’s exposure to atmospheric oxygen was limited, and we carried out the corresponding calculations. The decrease in the ARP indicator was calculated proportionally based on data for the cocoa powder itself, and for dark chocolate, converted to 100% cocoa powder in this product. The difference in values within the 95% confidence interval is 3.8–10.4%, a mean of 6.9% drop in the activity parameter for over 9 years of storage at room temperature, provided that oxygen access to the product is limited (i.e., the drop was mainly due to autoxidation of substances in the food system).

Thus, the addition of raw materials with high ARP values to candy and bakery products is an intuitive and correct technological solution. Examples of such raw materials include oils and lipid components (due to their polyunsaturated acids), vitamin preparations, hydrolysates (also for the production of functional products), and the grated nuts and seeds of plants. However, in some cases, extracts are preferable, primarily due to their more uniform distribution in the finished product.

The next food group we discuss is that containing cereals and legumes. The results are presented in Table 4.

We used extracts only from boiled cereals and legumes, since products in this category are subjected to heat treatment and are not used raw for food. As discussed above, the main categories of substances responsible for ARP are proteins and protein hydrolysates, lipids (including polyunsaturated ω-3 fatty acids), polyphenolic substances (including natural coloring components), and chlorophylls. In each food product, the contributions of these substances to the final ARP values are different and depend on their ratio in the raw material. However, the data obtained can easily explain the values from Table 4.

Low ARP values for rice cereals and chickpea beans can be explained by the high carbohydrate content; its contribution to ARP is rather small. Light-colored cereals, including amaranth and quinoa, do not contain highly colored substances, unlike, for example, buckwheat, lentils, or golden beans (whose shells contain chlorophyll and have a pronounced green color). The percentage of protein in the finished product decreases during cooking to 2–7%, or approximately 2–4 times the initial content by weight [36]; as a result, “minor” components such as lipids and coloring substances begin to make a significant contribution. In such cereals as chia and flax seeds, along with a high original protein content, there is also an increased content of ω-3 unsaturated fatty acids, which explains their position at the higher end of the table. Buckwheat and lentil groats also contain dyes, presumably of a polyphenolic nature, which increase their activity. Such high values for certain cereals and products of their processing must be taken into account when compiling new recipes for bakery and confectionery products, which, as has already become clear, do not have a high ARP.

For further discussion of vegetable raw materials, we consider Table 5. It shows data on the ARP levels of fresh and fresh-frozen fruits, vegetables, berries, and mushrooms.

The table indicates high values of ARP in vegetable raw materials, primarily due to the significant ascorbic acid content of fresh vegetables and fruits and the influence of anthocyanins (glycoside derivatives of quercetin and other polyphenolates) in cherries. Surprisingly, the average values for fresh Chinese cabbage are lower than those for fresh-frozen broccoli. 

We attribute this to several causes. The first is that, as fresh fruits and vegetables are transported, the act of breathing does not cease. This leads to waste from plastic substances being accumulated in leaves or roots, and raw materials selected from different harvesting periods differ significantly in this activity. The rate of destruction of plastic substances is apparently species-specific and also depends on the storage conditions of the raw materials (e.g., temperature and humidity) and requires a separate study. However, knowing that the production date of the Chinese cabbage in our study was approximately 3 weeks before its analysis, we can calculate the rate of ARP decline for it.

Chinese cabbage does not belong to the category of highly nutritious foods; it contains little protein, and the main substance it contains that has restorative properties is ascorbic acid (approximately 92% of the mass of all vitamins in the cabbage). Knowing the moisture content of the product, the concentration of vitamin C (from the USDA Nutrient Database), and the ratio of mass equivalents to discoloration, we calculate using molar masses the expected ARP value for a fresh cabbage, which is approximately 49–60 equivalents if we assume a linear dependence of the drop in values. Thus, the rate of decrease in the restorative properties during the storage of vegetables with a similar level of metabolism is approximately 4–18 equivalents per month.

The second reason that fresh Chinese cabbage’s ARP values are lower than those for fresh broccoli is that, during mass agricultural production, one-sided removal of nutrients from the soil occurs [9,37]. This is especially true for densely populated Asian countries and causes “soil depletion” by the crop, which implies a microelement deficiency in addition to the accumulation of pests in soil and plants [38]. The introduction of macronutrients in the form of fertilizers does not significantly improve this parameter of agricultural products.

Table 2 shows that some pure mineral components (e.g., selenic acid and its derivatives) may also exhibit ARP. However, in the case of “soil depletion”, the microelement level of agricultural raw materials falls, not only due to an insufficient concentration of substances but also due to the lower biochemical activity of tissue enzymes (both in plants and in animals that feed on these plants); as a result, they have less resistance to radicals.

A comparison of the ARP of mushrooms shows that dark-colored mushrooms have a higher value than light-colored ones. This is similar to what was noted for colored plants: the ARP is higher due to the higher concentration of polyphenol compounds they contain [39]. When comparing dried (since September 2017) and fresh-frozen mushrooms, extracts of the latter showed a greater activity by 4.9–5.1 times compared to the average values. Over a storage period of 3 years, this corresponds to a decrease in activity of up to 1.9 equivalents per month, with access to atmospheric oxygen at room temperature for coarsely ground raw materials.

Having considered the topic of plant processing, we now examine Table 6. It presents data on beverages, including juice, herbal drinks, and teas.

In Table 6, all but one of the samples (the carbonated drink) refer to drinks made from processed vegetable raw materials. Since the lowest ARP value is for the carbonated drink, we reasonably assume that other types of soda will have a similar level of activity. The basis of the recipe for sweet carbonated drinks is fast carbohydrates [40]. According to this author, they make up at least 95% of the mass of all dry matter in this product. As you can see in Table 3, refined sugar (and the caramel made from it) has one of the lowest ARP values we obtained in this study.

Juice products show increased activity values compared to carbonated drinks depending on two parameters: the percentage of juice in the drink and the type of coloring substances in the drink. Thus, slightly colored juice drinks (as well as juices) have a lower ARP value than drinks and juices made from colored raw materials [39]. For this reason, the activity increases in the row: apple < multi-fruit < grapes.

Next are drinks of natural origin: hibiscus, coffee, and tea. The average values for homemade hibiscus were slightly higher than those of hibiscus purchased in the retail network. Based on this data and the production time (May 2020), it is possible to estimate the rate of decline in ARP values for this type of product if we assume a linear dependence of the drop in values. It is 3.3–3.7% for 6 months, or 3.7 equivalents per month when stored at room temperature with limited access to atmospheric oxygen (the package contained a residual volume of gas).

Comparing the activity values of black and green tea, our data are consistent with those published earlier [41,42,43]. The drop in activity for black tea in relation to green tea is due to its longer fermentation and drying. Similar to tea, coffee also loses some of its components in direct proportion to the degree of roasting. Thus, green coffees, like green teas containing thermally labile vitamin C, have higher ARP than roasted or fermented ones [41]. Within the latter group, there is also an increase in potential for lightly-roasted and low-fermented coffee and tea varieties compared to more heavily processed ones [42,43]. Most likely, the effect of temperature reduces the ARP parameters in a similar way for other types of food raw materials [44]. We analyzed a medium-roasted coffee, which occupies an intermediate position in Table 6 between hibiscus and tea.

Based on the obtained ranges of values (tea has an ARP value 345 times higher than a carbonated drink), it is advisable to offer beverage manufacturers the opportunity to expand the introduction of new food recipes with extracts of coffee or tea, hibiscus or other edible plants, algae extracts, and amino acids. However, a separate study is required to assess the stability of amino acids in aqueous solutions. It is also highly advisable to enrich milk-based products (milk, milkshakes, etc.) with polyphenol compounds. ARP values for milk and products of its processing are presented in Table 7.

As noted above, during technological processing, there is usually a drop in activity. However, the opposite effect was recorded for milk; it was the only such product in the entire study. After boiling, the values increased by 4.8 times. We attribute this both to the partial hydrolysis of milk casein (with a change in protein conformation) and to the destruction of fat micelles of the milk emulsion, which contribute to a better interaction between radical particles and milk components. In this regard, we note that the fermentation of milk in the production of yogurt also increased the ARP value. We attribute this in part to the enzymatic activity of bacteria and to the fact that the caseins in yogurt have been degraded to smaller molecules; in addition, the amount of vitamins and organic acids from bacterial activity has increased.

The data obtained for the two types of cheese are partially consistent with this logic. One of them was subjected to a deeper enzymatic treatment with blue mold enzymes (Dorblu) that affected the ARP increase. In general, a trend towards a partial increase in ARP with the degradation of protein molecules in food systems can be traced. Nevertheless, other forms of fermentation, in our opinion, may not necessarily increase the potential values, as happens with teas. Perhaps, if tea were rich in protein, the “boiled milk” phenomenon would also occur.

In examining the next category of food products (Table 8), we first note the high activity in sausages. However, a more detailed study demonstrated that at least half of this activity is attributable to a physiologically toxic substance, namely sodium nitrite (NaNO_2_), which is added to meat products to give gray meat a pink or reddish color when interacting with methemoglobin [12,45]. Due to this bias, we did not investigate carbonates, frankfurters, sausages, or other products to which sodium nitrite could potentially be added. Average ARP values for meat products without sodium nitrite are not high and do not exceed 17 equivalents per gram of dry matter. 

ARP values for chicken meat and pork practically overlap within the 95% confidence interval, giving us reason to believe that other types of meat will show similar values. As we observed with other tables, heat treatment reduced the redox potential of chicken meat. We believe that there is a mixed trend. On the one hand, prolonged heat treatment contributes to the destruction of substances in the food system, and on the other hand, in products with an excess of water, it can slightly increase the values; this is due to the appearance of low-molecular-weight polypeptide molecules in solutions (due to hydrolysis) by analogy with hydrolysates from Table 2.

We note that eggs (a mixture of boiled protein and yolk) were not included in any of the tables, as they are represented in a separate group of products. Antiradical activity for this type of raw material was very low and amounted to 3.16–5.87 equivalents per gram of dry matter. We believe that these values may slightly increase in the case of egg avidin hydrolysis, but this requires additional experiments.

Due to the above-discussed influence of different groups of substances on food ARP, it can be assumed that terrestrial animals containing predominantly saturated fatty acids will have lower values of this parameter than, for example, marine fish species that store polyunsaturated fatty acids in the body. Table 9 presents data on the analysis of extracts from the tissues of fish, marine aquatic organisms, and products of their processing.

An initial analysis of the presented data reveals a large scatter in the values for all analyzed types of aquatic biological resources.

The highest values of the redox potential are registered for fish milt (salmon). A slightly lower value is shown for canned marine fish species, such as tuna and saury. An intermediate position is occupied by ARP values for shrimp, and the lowest values are registered for pollock extracts.

We divide the entire table into two groups and compare them. The first group is raw fish (primarily fresh-frozen fish), and the second group consists of products or semi-finished products that have undergone processing, such as minced meat (pollock), steam treatment (shrimp), heat treatment, and conservation. We compare the values in both of these groups. We begin by removing minced pollock from the calculation; we will discuss this point later. The average values for raw materials from marine fish species are in the range of 20–23 equivalents. Activity values for treated fish products range from 16 to 19 equivalents. The difference between the two ranges obviously indicates a drop in the ARP values during heat treatment, which can be traced for each type of raw material. The drop in average values for pollock is 12% (not significant, *p* > 0.05); for herring, 80% (significant, *p* < 0.05); for salmon and pink salmon, 27% (not significant for pink salmon, *p*> 0.05, however, if the trend persists, the value is significant for chum salmon, *p* < 0.05). For tilapia, it slightly increases by 8% (not significant, *p* > 0.05); for salmon milt, the drop is 57% (significant, *p* < 0.05).

The second item worthy of attention is the drop in the activity value for minced pollock. Taking into consideration that the value for fresh-frozen raw materials is statistically different from the values for frozen minced meat, and knowing the date of production (June 2020), we can estimate the rate of decline of the parameter for six months: 47%, or 0.35–0.45 equivalent per gram of dry weight per month for ground minced meat, glossy and stored at −18 °C in pressed parallelepipeds. If the trend continues, the ARP will be completely used up in approximately 12 months, which is contrary to the 18 months indicated on the packaging.

On the one hand, these data cause some confusion, but considering the lipophilicity of oxygen [21] and the experimental results indicating that cooling does not significantly reduce its solubility in the non-lipid fraction [29], the resulting values are not so unexpected. In this case, it is also necessary to take into account the fact that the grinding of raw materials introduces air bubbles into the volume of the product [46], with a simultaneous increase in the area of contact between gas and lipids, even at a lower temperature. Taking into account also the duration of contact with the source of oxidation (the gas introduced into the meat volume), we can explain why the glossing of this product is not an effective method for the preservation of ARP.

Raw materials from marine fish species (herring, pink salmon, chum salmon, saury, tuna, and navaga) and aquatic biological resources (shrimp) show a greater redox potential than fish grown on farms (tilapia). We believe that the reason is complex. On the one hand, it is known that there are more polyene fatty acids in marine fish. On the other hand, fish raised using aquaculture will accumulate only those components that they are fed, and most often these fodders are produced from the remains of raw materials. In other words, fish raised in aquaculture are to some extent similar to the cycle of plants grown in “soils with depletion” and may have low ARP values due to the weakness (imbalance) of their own food supply or the use of waste for their cultivation.

For comparison, we present Table 10, in which the activity values for some algae, grassy, and woody wild plants of northeast Russia are calculated. The analysis of these samples is dictated by the search for regionally specific raw materials that are available in Russia and have high ARP values.

As you can see, the ARP levels of extracts from plants grown in natural habitats significantly exceed those of all types of products (with the exception of NAC) discussed above. Thus, the maximum values obtained for willowherb exceeded even green tea by 33–35%. Extracts from small-flowered marigolds exceed, for example, the activity of medium-roasted coffee by 10–13%, and of hibiscus and all other juice-containing drinks by 15–16 times. On the other hand, this is not surprising, since quercetin derivatives (quercetin 3-O-glucuronide) with high parameters of anti-inflammatory and bacteriostatic action were found in willowherb extracts [47,48].

Taking into account the fact that the angustifolium fireweed can be found throughout the entire northern part of Russia and forms accumulations of large biomass, it seems possible to replace tea and tea raw materials with it. This is especially true in cases where only water or alcohol extracts are needed: in the production of beverages, and for adding to bakery, dairy, and confectionery products to increase the ARP of finished products.

No less promising is the use of extracts from pine and larch wood. They also form almost monospecific communities with a large biomass; this makes it possible to use extracts made from wood residues in food production more fully and rationally.

Fortunately, fireweed polyphenolic compounds show sufficient thermal stability. When the temperature regime of drying was deliberately exceeded (thus overheating the plant), the residual activity was still in the range of 29–30% within 4 h after the complete drying of the raw material.

The large biomass of these species, as well as high their ARP values, make it possible to recommend the use of coniferous extracts, their dried powders of wood and needles, algae thallus, and angustifolium fireweed for raw material replacement or inclusion in new food recipes. In our opinion, the use of herbaceous plants with colored flowers, such as marigolds, calendula, dandelion, milk thistle, etc., is also very promising. The use of such raw materials is important not only for imparting new aroma and taste qualities to products but also for the reparation of lost antiradical potential, as well as for increasing the shelf life and stability of food systems. 

## 4. Conclusions

In summary, we note that the parameter of antiradical activity is a comprehensive measure of the quality of food products and food raw materials. On the one hand, it is directly dependent on the initial concentration of vitamins, lipids, proteins, and other physiologically active components of food systems. On the other hand, through the Arrhenius constant and the law of mass action (including the partial pressures of gases), it is associated with environmental factors (such as temperature during storage and the concentrations of substances that affect the kinetics of chemical interaction), with methods of processing food raw materials, and with the technical parameters of the packaging. 

The analysis showed that a number of food products have very different values of ARP. For example, the products with the lowest level of this parameter included: hardtacks (3.05–3.40 eq.), monosodium glutamate (0.90–1.96 eq.), and caramel (2.26–3.26 eq.). On the contrary, the largest values were found for such products as: derivatives of cysteine such as NAC (7474.19–9166.13 eq.), an herbal infusion of willowherb from the Seymchan settlement (3313.95–3440.03 eq.), and green tea (2132.79–2265.9 eq.).

In addition to the factors mentioned above, the “strength” of counteracting both external oxidation factors and the internal self-oxidation of products directly depends on the ARP, which allows it to be used as an indirect indicator when setting the shelf life of consumer goods (under otherwise equal conditions).

It may also be expected that raw materials with high potential (including the addition of specific plant metabolites) will play an important role both in the development of functional foods and in the creation of specialized diets for medical reasons. 

## Figures and Tables

**Table 1:** The ARP for bakery products with a 95% confidence interval.

Product Name	Equivalents (95% CI)
Hemp hardtacks with the addition of hemp flour (Russian Federation)	3.05–3.40
Wheat bread “Baton” (long loaf) (Russian Federation)	4.02–5.34
Bread made from a mixture of rye and wheat flour (Russian Federation)	4.61–5.14
Bread with cranberries “Tayozhny” (Russian Federation)	8.48–8.93
Rye bread “Borodinsky” (Russian Federation)	18.53–21.70
**Mean value:**	**7.74–8.90**

**Table 2:** ARP of products widely used in the food industry, including biologically active substances, vitamins, hydrolysates, extracts, and mineral components.

Substance/Preparation Name	Equivalents (95% CI)
Monosodium glutamate crystalline (China)	0.90–1.96
Pea protein isolate (protein concentration 75%) (Great Britain)	14.37–16.48
Hydrolyzed whey protein (protein concentration 80%) (USA)	29.15–30.48
Soy protein isolate (protein concentration 90%) (Great Britain)	34.97–40.07
Arginine powder (USA)	52.33–65.65
Complex preparation of essential amino acid in powder Amino-9 (leucine, lysine, phenylalanine, valine, threonine, isoleucine, methionine, histidine, tryptophan) (USA)	93.9–106.7
Acetylcysteine powder (NAC) with Se (USA)	7474.19–9166.13
Complex vitamin preparation of tocopherols (the main α-form with an admixture of β- γ- δ-. without the addition of tocotrienols) (USA)	12.17–15.78
Soy lecithin (in terms of phosphatidylinositols in ≈20% by weight of lecithin) (USA)	16.3–17.8(81.72–88.90)
Conjugated Linoleic Acid—CLA (USA)	29.72–33.82
DHA+ EPA—docosahexaenoic and eicosapentaenoic acids in the ratio 2:1 (USA)	40.23–40.82
Chlorophyll extract liquid (in terms of pure chlorophyll) (USA)	92.01–100.48(1945.26–2203.45)
Lipoic Acid Powder (USA)	119.12–123.98
Selenic acid (Russian Federation)	300.50–306.30
Complex vitamin preparation B-100—B1; B2; B6; B12; niacin; folate; biotin; pantothenate (USA)	870.38–1390.46
Milk thistle bioflavonoids with the addition of extracts from dandelion and artichokes in a ratio of 6:2:1 (USA)	2728.16–3137.53
**Mean value:**	**706.84–847.77**

**Table 3:** ARP for candy products and some types of raw materials used in their production.

Product Name	Equivalents (95% CI)
Caramel made from pure beet sugar (own production)	2.26–3.26
“Darletto” soft waffles with whipped cream and blueberries (Russian Federation)	4.76–5.17
Pink chocolate with strawberry flavor (Belgium, Callebaut)	8.74–9.14
“Fruit nectar” Marmalade slices with mango flavor (Russian Federation)	9.25–9.69
“Druzhba” Halva with peanuts (Russian Federation)	9.32–9.57
Milk chocolate (Belgium, Callebaut)	11.61–11.81
“Babaevskiy” Dark chocolate (Russian Federation)	41.74–43.91
“Rossiyskiy” cocoa powder (Russian Federation)	52.46–54.47
Non-sulfated cane molasses (Paraguay, Wholesome Sweeteners Inc.)	44.68–47.58
**Mean value:**	**20.54–21.64**

**Table 4:** ARP values for cereals, legumes, the seeds of plants, and the products of their processing.

Product Name	Equivalents (95% CI)
Rice (Russian Federation)	7.96–23.61
Chickpea beans (India)	11.11–14.91
Amaranth (India)	14.20–23.12
Quinoa (Ecuador)	22.92–26.24
Lentil beans (India)	48.57–49.72
Ground flax seeds, fat-free (Russian Federation)	48.36–58.60
Buckwheat (Russian Federation)	63.91–67.25
Chia seeds (Mexico)	82.59–91.04
Mung beans (China)	98.04–113.33
**Mean value:**	**44.18–51.98**

**Table 5:** ARP values for plant raw materials (combined table, without division into subgroups).

Product Name	Equivalents (95% CI)
Dried mushrooms—*Leophyllum decastes* (Russian Federation)	17.39–18.65
Chinese cabbage, fresh (China)	46.34–57.28
Broccoli, fresh-frozen (Poland)	58.94–65.43
Fresh-frozen porcini mushrooms (Poland)	58.98–61.58
Fresh-frozen honey mushrooms (China)	74.30–84.97
Fresh-frozen champignons mushrooms (China)	130.92–132.25
Fresh Ligol apples (Republic of Moldova)	168.18–172.29
Cherry, fresh-frozen (Poland)	473.53–563.00
**Mean value:**	**128.10–144.21**
Mean value (for vegetable raw materials without mushrooms):	187.29–215.52
Mean value (only for mushrooms):	70.40–74.36

**Table 6:** Summary data on the analyzed extracts of teas, herbal, and juice drinks.

Product Name	Equivalents (95% CI)
Carbonated drink “Mangosteen” (Russian Federation)	6.49–6.55
Juice drink “Apple” (Russian Federation)	21.09–25.35
Juice drink “Multi-fruit” (Russian Federation)	37.43–37.89
Juice drink “Apple/grape” (Russian Federation)	57.09–60.94
Hibiscus drink (Egypt)	601.68–665.46
Hibiscus drink (Russian Federation)	645.61–666.50
Medium roasted “Coffee Jockey” (Russian Federation)	836.67–842.86
Black tea “Brooke Bond” (Netherlands)	2045.08–2134.17
Green tea “Greenfield Flying Dragon” (Russian Federation)	2132.79–2265.9
**Mean value:**	**709.32–745.07**

**Table 7:** ARP values for dairy products and milk.

Product Name	Equivalents (95% CI)
Raw cow’s milk (Russian Federation)	3.42–4.19
Cheese “Light” (Republic of Belarus)	4.92–8.12
Cheese “Dorblu” (Russian Federation)	8.07–10.35
Boiled cow’s milk (Russian Federation)	18.16–20.51
Yogurt without sugar, without fruit additives (Russian Federation)	54.07–65.02
**Mean value:**	**17.73–21.64**

**Table 8:** ARP values for meat and products of its processing.

Product Name	Equivalents (95% CI)
Canned food: stewed pork, without fat (Russian Federation)	10.60–12.08
Baked chicken meat (Russian Federation)	12.09–13.68
Fresh-frozen chicken meat (Russian Federation)	14.72–22.54
“Odesskaya” sausage (Russian Federation)	34.58–35.05
**Mean value:**	**18.00–20.84**
Mean value (without sausage):	12.47–16.10

**Table 9:** Data on ARP for products from fish, fish raw materials, and aquatic biological resources.

Product Name	Equivalents (95% CI)
Pollock mince, fresh-frozen (Russian Federation)	1.45–3.96
Canned herring (Russian Federation)	2.35–3.15
Pollock, boiled (Russian Federation)	4.11–5.04
Pollock, fresh-frozen (Russian Federation)	4.20–6.08
Canned pink salmon (Russian Federation)	6.22–12.51
Tilapia, fresh-frozen (Thailand)	9.31–13.72
Tilapia, boiled (Thailand)	11.55–13.50
Pink salmon, fresh-frozen (Russian Federation)	11.81–13.95
Pacific herring, fresh-frozen (Russian Federation)	12.36–14.69
Chum salmon, fresh-frozen (Russian Federation)	14.20–15.95
Salmon milt, boiled (Russian Federation)	19.33–21.21
Navaga, fresh-frozen (Russian Federation)	22.98–24.66
Northern shrimps, boiled, fresh-frozen (Denmark)	24.82–25.88
Canned saury (Russian Federation)	26.47–31.03
Canned tuna (Thailand)	27.13–30.20
Salmon milt, fresh-frozen (Russian Federation)	45.12–48.10
**Mean value:**	**15.91–18.54**
Mean value (for fresh-frozen raw materials)	15.32–17.88
(excluding pollock):	(20.32–23.02)
Mean value (for thermally processed products):	16.43–19.11

**Table 10:** The ARP level of extracts of some common wild plants and algae in the northeast of Russia.

Name of Wild Plants (*Latine*) and Production Parameters	Equivalents (95% CI)
Dahurian larch (*Larix gmelinii*), extracts from fine and coarse wood fractions	194.61–198.91
Extracts from powder preparations of brown, red, and green algae of the Sea of Okhotsk (genus: *Laminaria, Alaria, Ulva, Fucus, Chondrus, Neohypophyllum, Porphyra*). Average ARP values	213.2–237.81
Siberian dwarf pine (*Pinus pumila*), average values from extracts of dried needles, apical parts, and extracts from fresh samples	501.96–544.80
Rosebay willowherb, additional drying at 150 °C, 4 h (*Chamaenerion angustifolium*)	782.45–792.52
Marigolds (*Tagetes patula*), petal extract	931.12–978.24
Rosebay willowherb (*Chamaenerion angustifolium*), average values for extracts from leaves and flowers, drying at 60 °C	2611.12–2681.02
Rosebay willowherb (*Chamaenerion angustifolium*), local producers (Seymchan settlement, Magadan region)	3313.95–3440.03
**Mean value:**	**1221.20–1267.61**

## Data Availability

All data are presented within the article.
